# Does metal pollution matter with C retention by rice soil?

**DOI:** 10.1038/srep13233

**Published:** 2015-08-14

**Authors:** Rongjun Bian, Kun Cheng, Jufeng Zheng, Xiaoyu Liu, Yongzhuo Liu, Zhipeng Li, Lianqing Li, Pete Smith, Genxing Pan, David Crowley, Jinwei Zheng, Xuhui Zhang, Liangyun Zhang, Qaiser Hussain

**Affiliations:** 1 Institute of Resource, Ecosystem and Environment of Agriculture, Nanjing Agricultural University, Nanjing 210095-China; 2 Institute of Biological and Environmental Sciences, University of Aberdeen, 23 St Machar Drive, Aberdeen, AB24 3UU, UK; 3 Department of Environmental Science, University of California Riverside, CA 92521, USA; 4 Department of Soil Science and Soil Water Conservation, Pir Mehr Ali Shah Arid Agriculture University, Rawalpindi, Pakistan

## Abstract

Soil respiration, resulting in decomposition of soil organic carbon (SOC), emits CO_2_ to the atmosphere and increases under climate warming. However, the impact of heavy metal pollution on soil respiration in croplands is not well understood. Here we show significantly increased soil respiration and efflux of both CO_2_ and CH_4_ with a concomitant reduction in SOC storage from a metal polluted rice soil in China. This change is linked to a decline in soil aggregation, in microbial abundance and in fungal dominance. The carbon release is presumably driven by changes in carbon cycling occurring in the stressed soil microbial community with heavy metal pollution in the soil. The pollution-induced increase in soil respiration and loss of SOC storage will likely counteract efforts to increase SOC sequestration in rice paddies for climate change mitigation.

Soil respiration, leading to soil organic carbon (SOC) decomposition and CO_2_ efflux, is a major contributor to the increase in atmospheric CO_2_^1^. The rate of respiration was known to be influenced by soil temperature and moisture conditions^2^. Increased soil respiration, and thus SOC decomposition under warming, may be responsible for the release of carbon sequestered in the soil, referred to by Schulze and Freibauer^3^ as “unlocked carbon from soils”.

As a biogenic process, soil respiration is mediated by the soil microbial community and may be sensitive to changes in environmental factors. The role of soil microbial organisms in mediating of the release of biogenic greenhouse gases^4^ and in providing ecosystem productivity and services^5^ has been increasingly recognized. Metal pollution is becoming more widespread globally due to economic development and fast urbanization^6^. However, the understanding of soil microbial responses to heavy metal pollution is still very limited, though it is generally accepted that soil respiration is an indicator of microbial activity in heavy metal affected soils^7^. Heavy metal pollution may alter the ability of soils to retain carbon, by inducing changes in soil microbial community structure and activity^8^,^9^, in microbial abundance and diversity, in metabolic activity and thus in C utilization^10^.

Until now, evidence on the response of microbial respiration to metal pollution has been conflicting across studies. Whilst reduction in soil respiration, with a consequent reduction in SOM decomposition rate have frequently been observed in forest soils^11^, in experimentally spiked soils^12^, and in samples from contaminated fields in laboratory incubations^13^, very few field studies have suggested a significant influence^14^,^15^.

In China, agricultural soils have been shown to be extensively polluted with heavy metal. Rice paddies, mostly distributed in South China, are particularly affected by heavy metal pollution, causing a decline in grain yield and accumulation of toxic metals such as Cd, Pb and/or As in rice grains^16^. Whereas rice paddies constitute an important part of China’s SOC stock and contribute to C sequestration^17^, our present understanding of the impacts of heavy metal pollution on biogenic processes of C cycling and greenhouse gas emission in China’s rice paddies is still poor and based on a limited number of field studies.

Here, we report a significant increase in soil respiration and CO_2_ and CH_4_ evolution, and a concomitant reduction in topsoil C storage from a heavily metal-polluted rice paddy, compared to a nearby control site, from East China. This change is correlated with a significant decline in fungal abundance in the reduced microbial community, and decreased soil aggregation in the polluted soil. The study shows the sequestered carbon can be unlocked in metal polluted croplands, due to a modified soil C cycling within the stressed soil microbial community.

## Results

### Micro-aggregate size

As shown in Table 1, analysis of micro-aggregate size fraction distribution showed a decline by 39.4% in the share of large sized micro-aggregates (>0.2 mm) in the topsoil under heavy metal pollution.

### Microbial abundance and community structure

A significant reduction in the microbial community abundance and structure was observed in metal polluted soil (Table 2). A decline was seen in polluted (PF) over background (BG) field, by 22% in soil microbial biomass C and by 43% in total extractable microbial phospholipid fatty acids (PLFAs) (Table 2). Moreover, a marked shift in the soil microbial community was visible in PF plots with a 23% decrease in microbial C/N ratio together with a significant decline in fungal-to–bacterial ratio in PF over BG plots across the biological assays. This was shown by a 58% and 76% decline in cultivable organisms (Table S7), a 6.3% and 21% decrease in extractable PLFAs respectively in rice and wheat fields, and a 5% decrease in gene copy numbers in the rice field (Table 2).

### Soil respiration and CO_2_ emission

**Figure 1:**
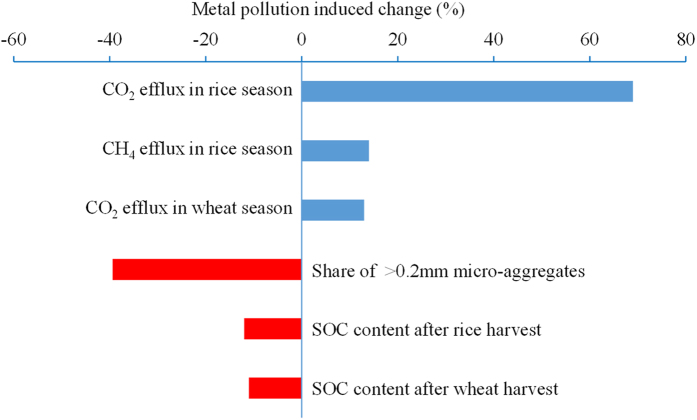
Metal induced changes (%) in soil respiration, micro-aggregate size fractions and topsoil organic carbon storage by comparing polluted plots to background plots. All the changes are significant at *p* < 0.05.

Soil respiration and CO_2_ emission rates were variable over the whole crop growing season (WCGS) but were consistent in showing that heavy metal pollution significantly increased respiration rates (Fig. 1). Soil CO_2_ and CH_4_ efflux across the WCGS was increased under pollution by 69% and 14% in the rice season, and soil CO_2_ efflux by 13% in the wheat season, respectively (Fig. 1, Table S1 and S2). There was an increase in soil basal respiration from PF plots compared to BG plots by 46% and 12% (Table S3 and S4), respectively under aerobic and anaerobic incubation for the topsoil samples from the wheat field and rice field during grain heading, and by 4% and 6% (Tabel S5 and S6) under aerobic incubation for the topsoil samples from the wheat field and rice field after harvest.

### SOC pool

Given soil respiration and CH_4_ emissions were significantly increased when soil was polluted by heavy metal, 12% and 11% net loss of topsoil SOC were observed in PF of rice field and wheat field compared to BG plots (Table 3). Meanwhile, microbial biomass C and N also showed a decrease in PF compared to BG.

## Discussion

Heavy metal pollution has been identified as a severe environmental issue for the potential hazards on environmental health and food safety. However, the knowledge of the effect of heavy metal pollution on SOC decomposition was limited. Zhang *et al.*^18^ resulted that heavy metal pollution increased basal respiration and qCO_2_ and decreased microbial biomass C and N in mining soils. Dumat *et al.*^19^ reported a decrease in SOC stock in croplands under multiple metals pollution. Being a main result of this study, marked increases in soil respiration and CO_2_ effluxes with heavy metal pollution were observed in a rice paddy. The increased respiration was accompanied by a concomitant 12% net loss of topsoil SOC in pollution field compared to background field. Accounting for 80% of the nation’s rice grain production, rice paddies had been increasingly polluted with heavy metals in major rice cultivation regions of China^20^,^21^. Heavy metal contamination in rice paddies could result in enhanced hazard for human health through soil-food chain transfer^22^. Given the decrease in SOC in polluted soil, heavy metal contamination may speed up climate change according to the results by this study. Meanwhile, a decrease in the proportion of large sized micro-aggregates was observed in polluted soils, which indicates a destruction of soil structure by heavy metal contamination. Given these, heavy metal contamination may cause multi-risks on food security, soil health and climate change.

Soil microorganism, which plays key roles in organic matter decomposition and nutrient cycling, is an important component of terrestrial ecosystems. There has been an increasing attentions evidenced that microorganisms are much sensitive to heavy metal stress in soils^18^,^23^. In this experiment, microbial biomass C significant decreased by 24% and by 22% in the field of rice season and wheat season, respectively. The reasons are possibly due to microorganisms in soil under heavy metal stress diverting energy from growth to cell maintenance functions^24^. Moreover, in heavy metal polluted soils, microorganisms need to exhaust more energy to survive in unfavorable conditions while they are stressed with metal toxicity, which is supported by the lower soil microbial quotient in polluted soils (Table 3). As a result of high metabolic quotient, a higher percentage of consumed carbon is released as CO_2_, and less C and N are built into organic components^25^. Data mentioned above could partly explain why soil respiration increased and SOC and SMBC decreased in metal polluted soil compared to background soil.

**Figure 2:**
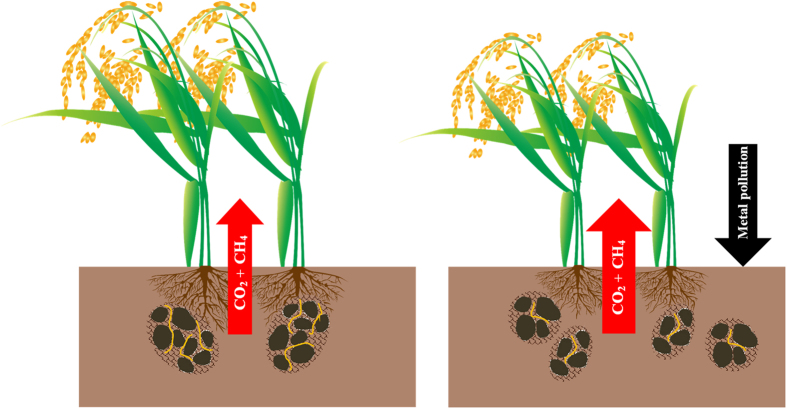
A proposed engine of soil C cycling modified in metal polluted rice paddy. The figure indicate the processes associated under metal stress, giving rise to an overall reduction in C storage with decline in crop productivity and thus increase in global warming potential (GWP) from rice production.

The decrease in SOC here is linked to a reduction not only in microbial abundance, but a decline in fungal-to-bacterial ratio in PF plots over BG plots (Table 2). This change is consistent with our previous finding of a cross site study of metal-polluted rice croplands from South China^26^. Cotrufo *et al.*^11^ reported a similar microbial community change with forest soils but with decreased respiratory C loss. Fungi-dominated microbial communities have been known to lower SOM decomposition rates in C cycling studies^8^,^27^, and in experiments with long term fertilization trails^26^ and with heavy metal affected soils^11^,^13^. In this study, nevertheless, the stressed microbial community change is characterized by a significant lower F/B ratio across different assays. Fungi in soil contribute to building-up large sized micro-aggregates^28^, and the reduction in cultivable fungi community could induce a decrease of large sized micro-aggregates, which has been observed in the polluted fields as shown in Table 1. As well known, soil organic matter can be physically protected with aggregates with the process inhibiting microbial access to the organic substrate^29^,^30^. Furthermore, large sized micro-aggregates play an important role in SOC sequestration, which have been shown in maize fields under tillage experiments from the USA^31^, and in rice paddies under long term organic/inorganic combined fertilization experiments^32^. This study indicates a marked decrease in the proportion of large sized micro-aggregates in heavy metal polluted soils (Table 1). Here, a combination of increased metabolic respiration and decreased physical protection could explain the overall increased soil respiration and in turn, the significant reduction in SOC storage due to enhanced SOC decomposition under metal pollution. Thus, we propose a modified engine as a mechanism for changes in biogenic processes of C cycling, with a decline in fungal dominance of the stressed soil microbial community driven by metal pollution in the rice soil (Fig. 2).

Extrapolating from the changes in CO_2_ efflux across WCGS, an increase in CO_2_ emission from PF plots over BG plots could amount to 0.2 t C ha^−1^ yr^−1^, over a whole rice/wheat rotation. This increase is significant to the estimated mean soil respiration rate of 1.7 t C ha^−1^ yr^−1^ 33. An annual SOC sequestration from rice paddies was reported in a range of 0.13–2.2 t C ha^−1^ yr^−1^ across mainland China^17^, and of 0.16 t C ha^−1^ yr^−1^ particularly for the Jiangsu Province^23^, where this study is located. Thus, the increased CO_2_ emission with metal pollution could potentially counteract the SOC sequestration in rice paddies, raising a critical challenge for food production and climate change mitigation in China’s rice agriculture.

In conclusion, metal pollution in the rice paddy has increased soil respiration and CO_2_ emissions with a concomitant decline in soil organic carbon storage. This is linked to a decline in the abundance of microbes and a reduction in fungal dominance of the stressed soil microbial community under metal pollution. This study highlights the need for serious consideration of metal pollution-induced changes in metabolic activity of decomposers in SOM stabilization and global C cycling modeling.

Thus, protection of paddy fields from heavy metal pollution, and restoration of those soils that are already polluted, could have a significant impact upon the ability of cropland soils to sequester carbon, as well as help them to sustain the high productivity essential to China’s food security.

## Methods

### Site and soil

The study site was in Yifeng Village, Xushe Township, Yixing Municipality (N 31° 24′, E 119° 41′), Jiangsu, China. The soil was a typical paddy soil, classified as a Fluvaquent. The local climate was a subtropical monsoon climate with a rainy and hot summer, and a cool and relatively dry winter. The soil has been cultivated with a summer rice-winter wheat rotation for a number of decades (SI). Since the late 1960’s, the area has experienced industrial development, and heavy metal pollution of multiple elements has occurred in part of the area adjacent to a metal smelter. From this area, the basic properties are similar, but the levels of metal accumulation of Pb, Cd Hg and As are divergent across plots. We used randomly selected polluted plots (downwind of the smelter) and relatively unpolluted ones (upwind of the smelter), with a distance of 600 m apart (SI), for a comparative study of soil respiration.

### Soil sampling and basic property measurement

Topsoil (0–15 cm) samples were collected in triplicate on each plot using an Eijkelkamp soil core sampler for lab incubation of basal respiration and basic properties as well as metal concentrations. For soil sample treatment, lab analysis of soil basic properties and metal contents, we followed the recommended protocols by Lu^34^ (SI). Undisturbed soil cores were untreated for soil aggregation analysis.

For basic properties, measurement and metal contents, samples were taken after wheat harvest, while soils for respiration testing and for microbial study were taken at different times with crop growth. For microbial studies, samples were collected with a stainless steel shovel in 5 random replicates to form a composite sample each plot. Samples were stored in sterilized closed plastic bags in an ice box for shipping and stored at 4 °C prior to incubation within 7 days.

### Chemical determinations

We measured SOC content using wet digestion with H_2_SO_4_-K_2_Cr_2_O_7_, titration with FeSO_4_ (SI); We performed digestions with a mixed acid solution of HF-HClO_4_-HNO_3_ (8:2.5:2.5, v:v:v), HCl-HNO_3_ (1:1, v/v), and HNO_3_-HClO_4_-HF (8:1:2, v/v/v) respectively for Cu, Pb, Cd, Zn, Cr, Ni, for As, Hg, and for Se. The contents in the digestions were determined with atomic absorption spectrophotometer (AAS) with atomic fluorescence spectrophotometer (AFS) for Hg, As and Se (SI). The measured soil properties and metal contents in given in Table 4 and 5.

### Field CO2 efflux measurement

We performed monitoring of soil CO_2_ efflux from soil respiration with a static closed chamber in triplicates on each plot, both for the polluted and control (background) fields in week interval during the whole crop growing season (WCGS) of a crop rotation year of rice and wheat. In each plot, three plastic flux collars (0.35 m × 0.35 m × 0.25 m) were permanently installed inter-rows over the whole annual cropping year. Gas was sampled during 9–11 AM over the rice season and during 1–3 PM over the wheat season. A gas sample was taken respectively at 0, 10, 20, and 30 min after chamber closure. Fluxes were determined from the slope of the mixing ratio change in these four samples. CO_2_ concentrations (and CH_4_ from rice field) of the gas samples were analyzed with a gas chromatograph (Agilent 7890 A) equipped with a flame ionization detector (FID) and an electron capture detector (ECD). Seasonal total flux of CO_2_ (and CH_4_ during rice growing) was sequentially accumulated from the emissions between every two adjacent intervals of the measurements. Soil temperature and moisture contents were also measured *in situ* with a Moisture Meter Type HH2. The setting of the chambers and the gas sampling and measurement is described in detail in SI.

### Measurement of basal respiration

Soil basal respiration was determined by lab incubation in a LRH-250-S incubator (Medicine Machinery Co. Ltd., Guangdong, China) at 25 °C ± 1.0 °C constantly for 28 days. We performed aerobic incubation consistently under water holding capacity (WHC) of 60% for samples both from the rice and wheat seasons, while anaerobic incubation constantly water submerged only for samples from the rice season. The incubations were done with 20 g of topsoil in a sealed jar and gas evolved in the headspace was collected every day during incubation course by syringe pressure. The gas concentration was determined with the same method as for field gas sample. The procedure was reported in a previous study^35^ and given in detail in SI. The soil basal respiration rate in a given time interval was calculated from the quantity of CO_2_ evolved, and normalized on the basis of the mean SOC contents of the sample (SI).

### Analysis of size fractions of soil micro-aggregates

Soil aggregation is much influenced by SOC content and fungal activity. We analyzed size fractions of soil micro-aggregates of undisturbed topsoil cores using a fractionation procedure with low energy dispersion developed by Stemmer *et al.*^36^ with minor modifications by Sessitsch *et al.*^37^. This procedure was reported in a previous study^35^ and given in detail in SI.

### Microbiological and biochemical analysis

We use a couple of standard microbiological assays (SI) to infer the changes in microbial community and structure with metal pollution. All the details are described in a recent work by Liu *et al.*^38^. Fresh samples (within 1 day of collection from the fields, See SI) were used for analyzing soil microbial biomass carbon and nitrogen, which was done with a fumigation and extraction procedure^39^. Microbial C content was determined with TOC analyzer (Jena MultiN N/C 2100, 2005), and N was determined with micro-Kjeldahl method. DOC was measured with a K_2_SO_4_ extraction and determined with TOC analyzer (Jena Multi N N/C 2100, 2005).

Culturable microbial population was analyzed with a procedure of dilute plate counting, basically following the procedure recommended by Zuberer^40^. Microbial phospholipid fatty acids (PLFAs) extraction and determination was performed for the same samples as for culturable organisms (SI), following a procedure described by Stemmer *et al.*^12^ (SI).

Total soil DNA was extracted with PowerSoil™ DNA Isolation Kit (Mo Bio Laboratories Inc., CA) according to the manufacturer’s protocol. Each DNA sample was amplified with F968 and R1401 set specifically for the bacterial community, and the NS1 and Fung-GC set specifically for the fungal community. With a real-time PCR (qPCR) assay of bacteria and fungi, the copy numbers of the bacterial 16S rRNA gene and the fungal internal transcribed spacer (ITS) rRNA gene in all the soil samples were determined in triplicate using an iCycler IQ5 Thermocycler (Bio-Rad, Hercules, CA). The quantification was based on the fluorescent dye SYBR-green one, which binds to double stranded DNA during PCR amplification. The primers and the thermal cycling conditions were as described by Fierer *et al.*^41^.

## Additional Information

**How to cite this article**: Bian, R. *et al.* Does metal pollution matter with C retention by rice soil? *Sci. Rep.* **5**, 13233; doi: 10.1038/srep13233 (2015).

## Supplementary Material

Supplementary Information

## Figures and Tables

**Table 1:** Soil micro-aggregate size fractions (%) of topsoil (0–15 cm). (Means ± S.D., n = 3, different lowercase characters in a same column indicate difference between polluted and background plot at *p* < 0.05).

Plot	**Size fractions**			
	**2–0.2** **mm**	**0.2–0.02 mm**	**0.02–0.002 mm**	**<0.002 mm**
Background	40.00 ± 0.87a	35.99 ± 1.33b	17.25 ± 1.44b	4.29 ± 0.30a
Polluted	24.23 ± 1.70b	41.45 ± 3.92a	23.22 ± 2.05a	5.63 ± 0.53a

**Table 2:** Microbial biomass (mg kg^−1^), culturable community of bacterial (10^7^ CFUs g^−1^) and of fungi (10^4^ CFUs g^−1^), total bacterial and fungal PLFA (nmol g^−1^), and gene copy number of bacterial (10^10^ g^−1^) and of fungi (10^8^ g^−1^) of topsoil from background and polluted soils respectively in rice and wheat season.

**Indicator**	**Rice season**		**Wheat season**	
	**Background**	**Polluted**	**Background**	**Polluted**
Biomass C	623.2 ± 35.2a	474.1 ± 15.6b	549.06 ± 18.23a	428.45 ± 11.82b
Biomass N	40.26 ± 1.87a	37.81 ± 2.49a	35.70 ± 1.87b	37.01 ± 2.49a
Culturable bacterial	6.47 ± 0.48a	6.98 ± 0.97a	5.77 ± 0.86b	12.30 ± 2.15a
Culturable fungal	16.64 ± 1.84a	7.48 ± 0.79b	6.51 ± 0.70a	3.27 ± 0.50b
Total PLFA	46.79 ± 3.06a	32.63 ± 3.49b	5.36 ± 0.03a	3.05 ± 0.03b
Bacterial PLFA	24.32 ± 1.62a	17.47 ± 1.68b	3.93 ± 0.70a	2.73 ± 0.38b
Fungal PLFA	8.59 ± 0.66a	5.78 ± 0.62b	0.31 ± 0.04a	0.17 ± 0.01b
Bacterial gene copy number	5.46 ± 0.82a	3.80 ± 0.44b	N. D^a^	N. D
Fungal gene copy number	14.3 ± 4.40a	9.50 ± 3.50b	N. D	N. D

(Means ± S.D., n = 3, different lowercase characters in a same row indicate difference between polluted and background plot at *p* < 0.05).

^a^Not determined

**Table 3:** Soil C pool (SOC), microbial biomass C (SMBC) and N (SMBN), and microbial quotient (SMBC/SOC) of the topsoil (0–15 cm).

	**Plot**	**SOC (g kg**^**−1**^)	**SMBC (mg kg**^**−1**^)	**SMBN (mg kg**^**−1**^)	**Microbial quotient (%)**
Rice season	Background	28.77 ± 1.11a	623.2 ± 35.2a	40.26 ± 1.87a	2.17 ± 0.03a
	Polluted	25.27 ± 0.53b	474.1 ± 15.6b	37.01 ± 2.49a	1.88 ± 0.03b
Wheat season	Background	28.23 ± 0.53a	549.06 ± 18.23a	35.70 ± 1.87b	1.94 ± 0.05a
	Polluted	25.11 ± 0.54b	428.45 ± 11.82b	37.01 ± 2.49a	1.70 ± 0.02b

(Means ± S.D., n = 3, different lowercase characters in a same column indicate difference between polluted and background plot at *p* < 0.05).

**Table 4:** Soil basic properties of topsoil (0–15 cm).

**Soil**	**pH (H**_**2**_**O)**	**Bulk density (g cm**^**−3**^)	**Total N (g kg**^**−1**^)	**Clay (g kg**^**−1**^)	**CEC (cmol kg**^**−1**^)
Background	6.94	1.38	2.80	317.5	16.34
Polluted	6.81	1.40	2.99	319.6	18.05

**Table 5:** Mean total concentration (mg kg^−1^) of heavy metals of topsoil.

**Filed**	**Hg**	**As**	**Cu**	**Zn**	**Pb**	**Cd**	**Cr**	**Ni**	**Se**
Background	0.36	4.46	34.48	33.81	45.84	0.66	23.44	13.22	0.68
Polluted	0.45	48.80	56.51	127.31	279.95	5.67	69.58	33.87	2.00
