# Supplementary material for: Does metal pollution matter with C retention by rice soil?
Source: Sci Rep. 2015 Aug 14;5:13233. doi: 10.1038/srep13233 (PMC4536517; doi:10.1038/srep13233)
Supplement: Supplementary Information [file srep13233-s1.pdf]

Supplementary Information (SI)

Does metal pollution matter with C retention by rice soil?

Authors: Rongjun Bian[1], Kun Cheng[1], Jufeng Zheng[1], Xiaoyu Liu[1], Yongzhuo Liu[1], Zhipeng Li[1], Lianqing Li[1], Pete Smith[2], Genxing Pan[1], David Crowley[3], Jinwei Zheng[1], Xuhui Zhang[1], Liangyun Zhang[1], Qaiser Hussain[1,4]

1, Institute of Resource, Ecosystem and Environment of Agriculture, Nanjing Agricultural University, Nanjing 210095-China;

2, Institute of Biological and Environmental Sciences, University of Aberdeen, 23 St Machar Drive, Aberdeen, AB24 3UU, UK;

3, Department of Environmental Science, University of California Riverside, CA 92521, USA;

4, Department of Soil Science and Soil Water Conservation, Pir Mehr Ali Shah Arid Agriculture University, Rawalpindi, Pakistan

Corresponding author: Genxing Pan

Address: Institute of Resource, Ecosystem and Environment of Agriculture, Nanjing Agricultural University, 1 Weigang, Nanjing 210095, China

Telephone/fax: +86 25 8439 6027

Email: pangenxing@aliyun.com; gxpan@njau.edu.cn

Additional Material and Methods

1. Site conditions

(1) ***Polluted plot*** (N 31 °24' 26", E 119 °41'36"), 400m downwind of a metal smelter,

(2) ***Unpolluted plot*** (N 31 °24'10", E 119 °41'28"), 600m upwind of the smelter.

The distance between the two sites is about 600 m

Soil type*:* Typical paddy soils with gley soil origin, same soil genesis for both plots. **A Stagnic Hydroagric Anthrosol** in Chinese Soil Taxonomy system; and **A Fluvaquent** in the US Soil Taxonomy System.

(3) ***Climate*****:** A typical monsoon climate in the northern subtropical zone, with hot and humid summers, and relatively dry and cool winters. Mean annual air temperature of 15.7 ℃, annual mean precipitation of 1177 mm during 1990-2005.

(4) ***Cultivation*****:** The paddy soil has been cultivated with rice and wheat for more than 15 years. Rice cultivation has been conducted on this soil for over one thousand years. In the recent 5years, the average yield per crop is 9 t ha^{-1} yr^{-1}.

2. Soil Sampling

Topsoil samples of 0-15 cm depth were sampled using Eijkelkamp soil sampling tool, one sample of about 0.5 kg is a composite of 3 subsamples collected around a same sampling point. Samples were taken in triplications in each plot.

3. Sample treatments

Samples were put in plastic bags and shipped to laboratory. For soil basic properties and soil basal respiration test, root free, air-dried at room temperature and ground to pass 2mm sieve samples were used. One portion of <2mm sample were further

ground to pass 0.25mm and 0.1 mm for soil pH (H_2O) and organic carbon, total nutrient pool and heavy metal content test. For microbial test, a portion of sample was stored at -20℃ in a refrigerator prior.

4. Soil basic property measurements

The performance for all these measurements was following the procedures described in Lu (2000) [1].

(1) ***Soil pH(H_2O)*****:** measured in a suspension of soil: water (CO_2 free) 1:2.5, determined with a Metteler -Toledo pH meter;

(2) ***Bulk density*****:** measured with a cylinder of 100cm^3 with fresh sample collected in field and weighted, the soil dry mass calculated after drying at 100℃ to constant weight of a portion of fresh sample;

(3) ***Soil total N*****:** measured by Kjeldahl method;

(4) ***Clay content*****:** measured by hydrometer in a suspension of 50g of gravel and root free soil in 250ml water, dispersed with 0.5 mol l^{-1} NaOH boiling on a thermal plate for 1h. After cooling down, the suspension decant to a sieve of 0.25mm, .and dilute to 1000ml. Stir of up and down for 1min and measure the specific weight after clay sedimentation in calculated time;

(5) ***CEC*** (cation exchange capacity): measured by leaching with 1 mol l^{-1} NH_4-AC-HAC buffer (pH 7) and NH_4^+ determined with the micro-Kjeldahl method.

5. Total content of heavy metals

(1) ***Digestion*****:**

Cu, Pb, Cd, Zn,Cr, Ni: mixed solution of HF-$HClO_4$-HNO_3 (10: 2.5:2.5, v/v/v) with varying volume ratio for different additions under 250℃ in a hot plate;

As, Hg: mixed solution of HCl-HNO_3 (1:1.v/v) in hot (over 90℃) water bath for 1h;

Se: mixed solution of HNO_3-$HC1O_4$-HF (8:1:2, v/v) under 165~175℃ in sand bath;

Digested Se reduced with HCl solution (1:1.v/v);

(2) ***Determination*****:**

Cu, Pb, Cd, Zn,Cr, Ni: atomic adsorption spectrophotometer (AAS) (TAS-986, Puxi General Instruments, Beijing) ;

As, Hg, Se: atomic fluorescence spectrophotometer (AFS) (AF-610, Bejing Ruili Instruments)

(3) ***Reference material:***

GSS-4 for Cu, Pb, Cd, Zn,Cr, Ni; GSS-2 for Hg and As, and GSS-4 for Se.

These reference materials for soils were bought from the China National Standard and Reference Material Service for use as internal standard with each batch of digestion.

(4) ***Recovery*****:**

Se: 93.3%~117.8%; Cd: 81.9~118.6%; Zn: 92.0~109.9%; Hg: 92.4~103.8 %; As: 91.4 ~105.5%;

Cu: 97.2~103.0 %; Pb: 85.5~96.5 %; Cr: 83.8~92.8%; Ni: 82.8~92.7%, respectively.

6. Soil respiration and CO_2 efflux measurements in field

(1) ***Chamber method***

We performed monitoring of soil CO_2 efflux from soil respiration with a static close chamber in triplicates each plot both of the polluted and background fields in a week

interval during the whole crop growing season (WCGS) of a crop rotation year of rice and wheat. In each plot, three plastic flux collars (0.35 m × 0.35 m × 0.25 m) were permanently installed inter-rows over the whole annual cropping year. The top edge of each collar had a groove (5 cm in depth) for filling with water to seal the rim of the chamber with a leveled surface. The chambers were made of aluminum and wrapped with a layer of sponge and aluminum foil to minimize air temperature changes inside the chamber during the sampling period. Each chamber was equipped with a circulating fan to ensure complete gas mixing and a temperature meter and a humidity meter installed inside the chamber. Gas sampled during 9-11AM over rice season and during 1-3 PM over wheat season. A gas sample was taken respectively at 0, 10, 20, and 30 min after chamber closure, fluxes were determined from the slope of the mixing ratio change in these four samples. Gas samples were shipped to lab for measurement within 12 hours. Soil temperature and moisture contents were also measured in situ with a Moisture Meter Type HH2. Simultaneous monitoring and gas sampling was done by 2 individual observers at the polluted and unpolluted plots respectively.

(2) ***Gas concentration determination***

CO_2 concentration (and CH_4 from rice field) of the gas sample was analyzed with a gas chromatograph (Agillent 4890D) equipped with a stainless steel column (Porapak Q) (80/100 mesh) and flame-ionization detector (FID). Column, injector and detector temperature were 35, 130 and 250 ℃, respectively. Nitrogen gas (carrier gas), FID hydrogen and FID airflow were set at the rate of 30, 45 and 400 ml min^{-1}, respectively.

Sample sets were rejected unless they yielded a linear regression value of r^2 greater than 0.90. Seasonal total flux of CO_2 (and CH_4 during rice growing) was sequentially accumulated from the emissions between every two adjacent intervals of the measurements. Soil respiration rate in a given time interval was calculated from the quantity of CO_2 evolved, and normalized on the basis of the mean SOC contents.

7. Soil basal respiration measurement with laboratory incubation

Soil basal respiration was determined using lab incubation in a LRH-250-S incubator (Medicine Machinery Co. Ltd., Guangdong, China) at 25 ℃ ± 1.0 ℃ constantly for 28 days. We performed aerobic incubation consistently under water holding capacity (WHC) of 60% for samples both from the rice and wheat seasons, while anaerobic incubation constantly water submerged only for samples from the rice season.

The procedure was as follows: 20g of samples (dry weight equivalent) were placed in a 120ml serum bottle and distilled water was added according to the incubation requirement. Each bottle was sealed with a rubber septum to allow gas sampling from the headspace. During incubation, a 0.25 ml sample of the gas evolved was collected every day by syringe pressure. For aerobic incubation, moisture content of the sample in the jar was adjusted with constant weight every day after gas sampling. For anaerobic incubation, the headspace of each bottle was flushed with N_2 gas (300 ml min^{-1}) for 10min before incubation. The slurries in each bottle were stirred for 3min using a magnetic bar before gas sampling, and then, the bottles were flushed with N_2 again (300 ml min^{-1}) for 10 min to keep an air of N_2 in the headspace. The Eh in the

slurry was monitored and measured with a Mettler-Toledo pH-Eh meter connected to a platinum electrode. Eh was maintained below −150 mV during the whole period of incubation. The CO_2 (and CH_4 under anaerobic incubation) concentration of the gas evolved was analyzed with a similar procedure as for the flux measurement. A blank of 40 ml distilled water was used as the control of the gas concentration in the bottle. The incubation was conducted in triplicate bottles.

The gas concentration was determined with the same method as for field gas sample. The procedure was reported in a previous study[2]. The C mineralization rate in a given time interval was calculated from the quantity of CO_2 produced, and normalized on the basis of the initial SOC contents of the sample.

8. Soil C pool determinations

(1) *Total organic carbon*

Total organic carbon content was measured using wet digestion with H_2SO_4-$K_2Cr_2O_7$, titration with $FeSO_4$.

(2) *Microbial biomass C, DOC and labile C*

Measurements were done using fresh samples within 1 day after collection from the field. Microbial biomass carbon was determined using a fumigation and extraction procedure[3]. C determined with TOC analyzer (Jena MultiN N/C 2100, 2005); N determined with micro-Kjeldahl method. DOC was measured with an extraction by K_2SO_4 and determined by TOC analyzer (Jena Multi N N/C 2100, 2005). Labile carbon was measured by oxidation with a solution of 0.33 mol L^{-1} potassium permanganate ($KMnO_4$)[4].

9. Microbial community structure analyses

(1) *Culturable population size analysis*

Samples:

Samples collected in 5 replications of randomly selected locations (each is a composite of 3 subsamples) both from rice and wheat-growing field during grain filling stage. The samples were taken with a stainless steel shovel; Samples were stored in sterilized closed plastic bags in an ice box for shipping and stored at 4℃ prior to incubation within 7days at laboratory.

Incubation procedure

Population analysis by dilute plate media incubation and counting of colonies was performed basically following the procedure recommended by Zuberer (1994)[5].

The plate counting of culturable microorganisms was performed as following procedures. Soil sample (1 g) was suspended in sterile distilled water to form a decimal dilution up to 10^{-5}. An aliquot of 50 or 100 μl of the diluted suspension of 10^{-4} or 10^{-5} was spread on a beef-protein medium plate in Petri dish, and an aliquot of 50 μl at 10^{-2} dilution on a Martin's medium plate for counting of bacteria and fungi, respectively. Control plates of respective media without soil suspension were also included to check any possible contamination. The colonies on each medium plate were counted after inoculation at 28 ℃. Plates that carried 10 to 100 colonies (fungi) were counted on day 2 and that carried 30 to 200 colonies (bacteria) were counted on day 4. The Colony Forming Units (CFUs) per gram of dry soil was calculated. The operation of the individual plate incubation and counting was done in triplicates.

(2) ***Media for incubation:***

Bacteria: Beef extract-Peptone medium;

Actinomycetes: Gause 1 medium, add 0.1ml 5% sterilized potassium permanganate solution per 300-mL medium prior to incubation;

Fungi: Martin's medium.

All the media were sterilized using autoclavation at 121℃ for 20 min

(3) ***Phospholipid fatty acids analysis (PLFA)***

Samples

Samples were collected in triplicates of randomly different locations (each is a composite of 5 subsamples) from rice field after rice harvest in October, 2007. The samples were taken with a stainless steel shovel; Samples were stored in sterilized closed plastic bags in an ice box for shipping and frozen at -20℃ prior to extraction within 7 days;

Extraction of microbial PLFAs

Microbial phospholipid fatty acids were extracted using the modified procedure of Bligh-Dyer[6] as described by Kehrmeyer et al. (1996)[7]. Briefly, 5 g of frozen-dry soil sample was extracted with a mixture of chloroform–methanol -phosphate (1: 2: 0.8), and the lipids were separated into neutral lipids, glycol-lipids, and polar lipids[8,9] on a pre-packed silicon acid column (Ultra-CleanTM, 500 mg/4ml NH-2 SPE columns, Alltech, Inc.). The polar lipid fraction was trans-esterified with a mild alkali solution to recover the PLFA as methyl esters in hexane.

PLFA s determination

This analysis was done in the Laboratory of Life Science, Nanjing University. The PLFA obtained was dissolved with a reagent (MiDI 50/50% Hexane: Methyl tert-Butyl Ether) and analyzed with a gas chromatography (GC, HP 6890 Series, Hewlett Packard, Wilming-ton, Del.). The fatty acid 19:0 was added as an internal standard and the PLFAs were identified using MIDI 4.5 peak identification software (MIDI, Inc., Newark, DE, 2002). The total PLFAs concentration was expressed as nmol g^{-1} soil on a dry weight basis.

(4) *PCR-DGGE protocol*

DNA extraction

Total DNA was extracted with PowerSoil™ DNA Isolation Kit (Mo Bio Laboratories Inc., CA) according to the manufacturer's protocol.

PCR-DGGE analysis of bacterial and fungal community

Each DNA sample was amplified with F968 and R1401 set specific for the bacterial community[10], and the NS1 and Fung-GC set specific for the fungal community[11]. The GC clamp described by Muyzer et al. (1993)[12] was added to the 5′ end of a primer to stabilize the melting behavior of the DNA fragments. PCR reaction was performed in an Eppendorf autothermer Cycler (Bio-Rad) using 25 µl reaction volume. The reaction mixture contained 12.5 µl Go Taq® Green Master Mix (Promega), 1 µl of 10 µM of each primer, 9.5 µl of sterile ddH_2O and 1 µl of DNA template. For DGGE analysis, PCR products were separated on 8% w/v polyacrylamide gels [acrylamide–bisacrylamide (37.5: 1)] containing denaturing gradients of 40–60% for bacteria and 20–40% for fungi using the Bio-Rad D-Code universal mutation

detection system. A 100% denaturant was defined as 8% acrylamide containing 7 M urea and 40% deionized formamide. DGGE was performed using 20 μl (bacteria) and 11 μl (fungi) of the PCR product in 1 × TAE buffer at 60 ℃, 200 V for 5 min and then 140 V for 500 min (bacteria) and 100 V for 540 min (fungi). Gels were stained with silver staining[13], and then the gels were photographed with Gel Doc-2000 Image Analysis System (Bio-Rad, USA). Digitized DGGE images were analyzed with Quantity One image analysis software (Version 4.0, Bio-Rad, USA). This software identifies the bands occupying the same position in the different lanes of the gel. A band of DNA was detected if it accounted for greater than 1% of the total lane intensity.

(5) Real-time PCR (qPCR) assay of bacteria and fungi

The copy numbers of the bacterial 16S rRNA gene and the fungal internal transcribed spacer (ITS) rRNA gene in all the soil samples were determined in triplicate using an iCycler IQ5 Thermocycler (Bio-Rad, Hercules, CA). The quantification was based on the fluorescent dye SYBR-green one, which binds to double stranded DNA during PCR amplification. The primers and the thermal cycling conditions were as described by Fierer et al. (2005)[14]. The DNA concentration of all soil samples was measured at 260 nm using a UV Spectrophotometer (Bio Photometer, Eppendorf, Germany) and then adjusted to 15 ng μl^{-1}. Each reaction was performed in a 25 μl volume containing 15 ng of DNA, 1 μl of 10 μM of each primer and 12.5 μl of SYBR premix EX Taq TM (Takara Shuzo, Shinga, Japan). Melting curve analysis of the PCR products was conducted following each assay to confirm that the

fluorescence signal originated from specific PCR products and not from primer-dimers or other artifacts. PCR products were checked for the correct size by comparison to a standardized molecular weight ladder by electrophoresis on 1.5% agarose gel.

A plasmid standard containing the target region was generated for each primer set using total DNA extracted from the soil samples. The amplified PCR products of bacterial 16S rRNA gene and the fungal ITS rRNA gene were purified using PCR solution purification kit (Takara), ligated into pEASY-T3 cloning vector (Promega, Madison,WI) and cloned into *Escherichia coli* DH5α. Clones containing correct inserts were chosen as the standards for qPCR. Plasmid DNA was isolated using plasmid extraction kit (Takara) and DNA concentrations were determined by spectrophotometer as mentioned above. As the size of the vector and PCR inserts were known, the copy numbers of the 16S rRNA gene and the ITS rRNA gene were directly calculated from the concentration of extracted plasmid DNA. Standard curves were generated using triplicate 10-fold dilutions of plasmid DNA ranging from 3.72×10^2 to 3.72×10^8 copies for the bacterial 16S rRNA gene, and 1.09×10^2 to 1.09×10^8 copies of template for the ITS rRNA gene per assay. High amplification efficiencies of 111% were obtained for the bacterial 16S rRNA gene and 95.3% for fungal ITS rRNA gene quantification, with R^2 values between 0.992 and 0.995 and slopes from -3.08 to -3.44, respectively. The relative fungal/bacterial ratio was calculated as the ratio of copy numbers measured using the ‘total fungi’ and ‘total bacteria’ qPCR assays[14].

10. Measurements of soil micro-aggregate size fraction distribution

The fractionation procedure was based on the method developed by Stemmer et al. (1998)[15] with minor modifications[16]. A fresh soil was dispersed in distilled water by use of a probe-type ultrasonic dis-aggregator (Shanghai Zhixin, JVD-650) with output energy of 0.2 kJ g^{-1} for 5min. The fraction of 2-0.2mm was collected in sieves with a corresponding mesh size. The fraction of 0.2-0.02mm was obtained by sedimentation after siphonage. The remainder was centrifuged to collect the fraction of 0.02-0.002mm and the supernatant was centrifuged to collect the fraction of <0.002mm. All the separates of size fractions obtained were oven-dried and weighted .The recovery of the fractions to the total mass was in a range of 95%-98%. Fresh soil was dispersed in distilled water by use of a probe-type ultrasonic disaggregator (Shanghai Zhixin, JVD-650) with output energy of 0.2 kJ/g for 5min. The fraction of 200–2000 μm was collected in sieves with a corresponding mesh size. The fraction of 20–200μm was obtained by sedimentation after siphonage. The remainder was centrifuged to collect the fraction of 2–20 μm and the supernatant was centrifuged to collect the fraction of <2 μm. The fraction separates were freeze-dried prior to the analysis.

11. Statistics and significance of difference test

Data treatment was performed with Microsoft EXCEL 2013. Test of significance of difference between polluted and background soils was done with students TEST. Significance was defined at $p<0.01$ or $p<0.05$. Statistical analyses were performed using the SPSS16.0 (SPSS Inc., Chicago, IL, USA).

Results

Table S1 Soil CO_2 efflux measurement (mg CO_2-C m^{-2} d^{-1}) and temperature condition (℃) during rice season

Date		25-Jul	1-Aug	8-Aug	15-Aug	24-Aug	31-Aug	7-Sep	14-Sep	21-Sep	28-Sep	4-Oct	11-Oct	16-Oct	Total
Back-	1	550.8	547.1	782.3	1451.1	1240.5	1524.1	1225.7	1940.9	1451.2	2695.3	2903.6	3031.4	1982.2	149.3
ground	2	664.5	597.1	627.6	1768.7	1421.0	1238.6	1495.3	1677.4	1497.5	2390.2	2470.8	2878.7	1619.9	142.4
	3	597.9	712.5	684.1	1636.9	1469.0	1513.9	1396.7	1893.5	1225.4	2769.6	2627.9	2603.7	1552.7	144.8
	Soil	30.1	29.5	29.0	26.5	29.1	26.7	26.5	21.1	20.8	24.0	24.2	21.5	21.6	
	Box	32.1	33.3	32.1	27.3	30.2	28.1	27.5	23.6	22.8	25.3	25.5	21.8	22.0	
Polluted	1	1277.0	1353.2	1822.3	3289.7	2171.0	3559.2	2812.7	3332.0	2939.9	3427.0	3417.8	5409.6	2743.4	262.9
	2	1209.3	1094.6	1529.0	2697.8	1806.5	3098.6	2868.2	2865.3	2280.9	2822.2	2936.3	5315.4	3127.4	235.6
	3	1016.2	1233.5	1650.8	3242.3	1948.2	2881.7	3581.5	2723.7	2274.2	3066.3	3398.3	4737.4	2512.5	239.9
	Soil	29.8	29.2	28.7	26.7	29	26.8	26.5	21	20.6	23.7	24.1	21.2	21.7	
	Box	32.5	33.5	32	27.5	30	28	27.3	23.5	23	25	25.4	22	21.8	
Air temperature		33.8	35.1	33	27.5	30.1	27.8	27.2	23	22.7	26	26.5	22.3	22.2	

Table S2 Soil CO_2 efflux measurement (mg CO_2-C m^{-2} d^{-1}) and temperature (℃)condition during wheat season

Date of measurement		28-Mar	4-Apr	11-Apr	18-Apr	25-Apr	2-May	10-May	18-May	25-May	Total
Background	1	431.5	552.8	821.9	1123.4	1305.1	1503.2	1802.5	1732.0	1710.5	76880.3
	2	454.7	571.6	829.0	1018.4	1151.4	1298.6	1618.3	1865.3	1793.7	74207.3
	3	517.9	530.1	850.3	1139.1	1348.2	1331.7	1681.2	1723.7	1621.3	75204.4
	Soil	12.1	11.5	13.4	14.6	15.1	15.0	15.6	15.4	15.6	
	Box	14.5	13.0	14.6	20.7	21.8	22.2	21.8	22.4	22.5	
Polluted	1	473.8	577.1	892.3	1262.8	1340.5	1624.1	1825.7	1942.9	2093.3	84227.1
	2	509.1	587.1	827.6	1318.4	1421.0	1438.6	1795.3	2068.4	1987.7	83672.6
	3	516.2	661.5	921.1	1446.2	1569.3	1712.1	1932.7	1793.1	1817.1	86585.2
	Soil	12.0	11.5	13.5	14.5	15.2	15.0	15.7	15.4	15.8	
	Box	14.6	12.7	14.8	20.8	22.0	22.1	21.9	22.3	22.6	
Air temperature		15.1	13.2	15.2	21.5	25.1	25.2	25.2	25.6	25.8	

Table S3 Soil basal respiration rate (mg CO_2-C g^{-1} OC d^{-1}) from the wheat field at heading stage under aerobic incubation at 25℃

Day of incubation		1	2	3	4	5	6	7	8	9	10	11	12	13	14
Background	0-15cm	2.26	2.31	1.59	1.30	0.87	0.75	0.55	0.59	0.50	0.49	0.41	0.44	0.35	0.36
		2.95	2.67	1.89	1.44	1.01	0.66	0.57	0.66	0.52	0.55	0.48	0.43	0.39	0.41
		2.38	2.36	1.72	1.35	0.93	0.70	0.40	0.67	0.47	0.51	0.43	0.42	0.38	0.38
Polluted	0-15cm	3.84	3.59	2.91	2.47	1.64	1.48	1.34	1.23	1.04	0.99	0.58	0.61	0.59	0.54
		3.79	3.88	3.12	2.69	1.45	1.24	1.13	0.96	0.93	0.83	0.57	0.51	0.54	0.50
		3.06	2.66	2.05	1.72	1.14	1.01	0.75	0.89	0.73	0.69	0.51	0.49	0.44	0.40

Day of incubation		15	16	17	18	19	20	21	22	24	25	26	27	28
Background	0-15cm	0.38	0.34	0.29	0.28	0.34	0.34	0.25	0.22	0.34	0.30	0.25	0.25	0.23
		0.40	0.34	0.33	0.32	0.39	0.29	0.29	0.26	0.38	0.38	0.28	0.32	0.28
		0.40	0.36	0.32	0.29	0.35	0.27	0.28	0.23	0.36	0.31	0.29	0.31	0.26
Polluted	0-15cm	0.64	0.51	0.49	0.45	0.52	0.43	0.38	0.31	0.50	0.45	0.38	0.38	0.34
		0.59	0.48	0.47	0.44	0.51	0.42	0.36	0.30	0.48	0.49	0.40	0.40	0.34
		0.46	0.39	0.35	0.33	0.39	0.31	0.30	0.22	0.39	0.09	0.09	0.36	0.30

Table S4 Soil basal respiration rate (mg CO_2-C g^{-1} OC d^{-1}) from rice field at heading stage under anaerobic incubation at 25 ℃

Day of Incubation		2	4	6	8	10	12	14	16
Background	1	0.84	0.69	0.53	0.38	0.33	0.46	0.28	0.40
	2	0.88	0.63	0.53	0.37	0.44	0.41	0.27	0.42
	3	0.80	0.63	0.51	0.39	0.36	0.45	0.27	0.40
Polluted	1	1.02	0.71	0.56	0.50	0.59	0.54	0.45	0.56
	2	0.93	0.74	0.51	0.44	0.63	0.56	0.45	0.60
	3	0.95	0.76	0.56	0.50	0.66	0.48	0.43	0.55

Day of incubation		19	22	25	28	31	34	37	40	43
Background	1	0.29	0.35	0.35	0.36	0.38	0.27	0.34	0.24	0.15
	2	0.32	0.36	0.34	0.34	0.36	0.26	0.35	0.25	0.21
	3	0.29	0.36	0.34	0.34	0.36	0.27	0.35	0.25	0.19
Polluted	1	0.43	0.56	0.41	0.41	0.50	0.30	0.36	0.34	0.25
	2	0.39	0.53	0.39	0.41	0.54	0.34	0.35	0.33	0.26
	3	0.44	0.55	0.43	0.39	0.46	0.36	0.36	0.29	0.24

Table S5 Soil basal respiration rate (CO_2-C g^{-1} OC d^{-1}) from the wheat field after wheat harvest under aerobic incubation at 25℃

**Day of incubation**	**0.5**	**1**	**1.5**	**2**	**3**	**4**	**5**	**6**	**7**	**8**	**9**	**10**	**11**	**12**	**13**
Background	2.59	1.71	1.34	1.22	1.16	1.04	0.56	0.37	0.25	0.28	0.33	0.26	0.27	0.25	0.19
	2.62	1.70	1.42	1.20	1.15	1.09	0.58	0.37	0.32	0.29	0.22	0.25	0.24	0.24	0.21
	2.71	1.72	1.45	1.26	1.20	1.02	0.54	0.38	0.32	0.28	0.28	0.26	0.23	0.24	0.19
Polluted	2.47	1.60	1.32	1.09	1.23	1.21	0.65	0.48	0.50	0.42	0.38	0.31	0.33	0.32	0.19
	2.35	1.59	1.31	1.08	1.22	1.12	0.62	0.51	0.50	0.36	0.33	0.29	0.29	0.29	0.22
	2.41	1.62	1.39	1.15	1.28	1.16	0.70	0.47	0.42	0.32	0.34	0.34	0.31	0.28	0.22

**Day of incubation**	**14**	**15**	**16**	**17**	**18**	**19**	**20**	**21**	**22**	**23**	**24**	**25**	**26**	**27**	**28**
Background	0.20	0.15	0.14	0.15	0.14	0.15	0.14	0.17	0.16	0.14	0.16	0.16	0.16	0.16	0.15
	0.19	0.13	0.13	0.14	0.15	0.14	0.15	0.15	0.14	0.15	0.15	0.14	0.13	0.13	0.13
	0.18	0.13	0.14	0.15	0.14	0.15	0.14	0.17	0.16	0.14	0.16	0.15	0.14	0.14	0.14
Polluted	0.19	0.18	0.16	0.15	0.14	0.14	0.17	0.15	0.17	0.14	0.15	0.14	0.15	0.13	0.13
	0.19	0.19	0.17	0.15	0.15	0.18	0.17	0.17	0.15	0.15	0.17	0.15	0.16	0.15	0.15
	0.20	0.16	0.18	0.16	0.16	0.16	0.15	0.18	0.15	0.15	0.16	0.14	0.13	0.13	0.13

Table S6 Soil basal respiration rate (CO_2-C g^{-1} OC d^{-1}) from the rice field after rice harvest under aerobic incubation at 25℃

Day of incubation	0.5	1	1.5	2	3	4	5	6	7	8	9	10	11	12	13
Background	2.51	2.05	1.65	1.46	1.18	1.16	0.72	0.46	0.33	0.34	0.37	0.31	0.28	0.30	0.20
	2.62	1.97	1.72	1.50	1.25	1.19	0.68	0.47	0.32	0.32	0.35	0.32	0.30	0.31	0.21
	2.71	1.92	1.75	1.56	1.32	1.22	0.64	0.48	0.32	0.31	0.34	0.33	0.32	0.29	0.19
Polluted	2.15	2.01	1.62	1.41	1.66	1.34	0.87	0.65	0.38	0.37	0.37	0.36	0.36	0.35	0.22
	2.37	1.89	1.71	1.38	1.62	1.42	0.92	0.61	0.40	0.36	0.37	0.36	0.36	0.36	0.22
	2.41	1.76	1.79	1.35	1.58	1.46	1.00	0.57	0.42	0.35	0.37	0.36	0.37	0.36	0.22

Day of incubation	14	15	16	17	18	19	20	21	22	23	24	25	26	27	28
Background	0.18	0.12	0.14	0.15	0.13	0.15	0.15	0.15	0.15	0.14	0.15	0.14	0.13	0.13	0.13
	0.19	0.13	0.13	0.14	0.15	0.14	0.15	0.15	0.14	0.15	0.15	0.14	0.13	0.13	0.13
	0.20	0.13	0.14	0.15	0.14	0.15	0.14	0.17	0.16	0.14	0.16	0.15	0.14	0.14	0.14
Polluted	0.18	0.17	0.17	0.15	0.17	0.17	0.18	0.16	0.16	0.16	0.17	0.15	0.15	0.15	0.14
	0.19	0.17	0.17	0.15	0.16	0.17	0.18	0.17	0.17	0.17	0.16	0.15	0.15	0.15	0.15
	0.20	0.16	0.17	0.15	0.15	0.16	0.15	0.15	0.15	0.15	0.16	0.15	0.14	0.14	0.14

Table S7 Microbial population size of the cultivable microorganisms. (Means ± S.D., n=3, different lowercase characters in a same column indicate difference between polluted and background plot at *p*<0.05)

	Field	Actinomycetes (10^6 CFUg^{-1} soil)	Bacteria (10^7 CFUg^{-1} soil)	Fungi (10^4 CFUg^{-1} soil)	F/B Ratio (× 10^{-3})	B/A ratio (× 10^{-1})
Rice season	Background	4.81±0.43a	6.47±0.48a	16.64±1.84a	2.57±0.12a	1.35±0.13a
	Polluted	3.26±0.60b	6.98±0.97a	7.48±0.79b	1.17±0.05b	2.16±0.55a
Wheat season	Background	2.88±0.26a	5.77±0.86b	6.51±0.70a	1.13±0.06a	1.99±0.12b
	Polluted	1.34±0.11b	12.30±2.15a	3.27±0.50b	0.27±0.05b	9.16±0.87a

Samples of topsoil (0-15cm) collected respectively from rice and wheat fields after crop harvest. F/B ratio: Population of fungi divided by that of bacteria; B/A ratio: population of bacteria divided by that of Actinomycetes; Different capital and low case characters in a same column mean difference between polluted and Background plot at $p<0.05$ respectively.

References:

1. Lu, R. K. Methods of Soil and Agro-chemical Analysis. Beijing: China Agric Sci Tech Press, Beijing (2000).
2. Zheng, J., Zhang, X., Li, L., Zhang, P. & Pan, G. Effect of long-term fertilization on C mineralization and production of CH_4 and CO_2 under anaerobic incubation from bulk samples and particle size fractions of a typical paddy soil. *Agric. Ecosyst. Environ.* **120**, 129–138 (2007).
3. Vance, E. D., Brookes, P. C. & Jenkinson, D. S. An extraction method for measuring soil microbial biomass C. *Soil Biol. Bioche*. **19**, 703-707 (1987).
4. Blair, G. J., Lefroy, R. D. & Lisle, L. Soil carbon fractions based on their degree of oxidation, and the development of a carbon management index for agricultural systems. *Crop Pasture Sci*. **46**, 1459-1466 (1995).
5. Zuberer, D. A. Recovery and Enumeration of Viable Bacteria. In *Methods of Soil Analysis: Part 2-Microbiological and Biochemical Properties*. (eds Weaver, R. W. *et al.*) 119-144 (Soil Science Society of America, 1994).
6. White, D. C., Davis, W. M., Nickels, J. S., King, J. D. & Bobbie, R. J. Determination of the sedimentary microbial biomass by extractible lipid phosphate. *Oecologia* **40**, 51-62 (1979).
7. Kehrmeyer, S. R. et al. Combined lipid/DNA extraction method for environmental samples. *J Microbiol. Methods* **25**, 153–163 (1996).
8. Guckert, J. B., Antworth, C. P., Nichols, P. D. & White, D. C. Phospholipid, ester-linked fatty acid profiles as reproducible assays for changes in prokaryotic

community structure of estuarine sediments. *FEMS Microbiology Ecol.* **31**, 147-158 (1985).

9. MacNaughton, S. J. et al. Microbial population changes during bioremediation of an experimental oil spill. *Appl. Environ. Microb.* **65**, 3566-3574 (1999).
10. Heuer, H., Krsek, M., Baker, P., Smalla, K. & Wellington, E. M. Analysis of actinomycete communities by specific amplification of genes encoding 16S rRNA and gel-electrophoretic separation in denaturing gradients. *Appl. Environ. Microb.* **63**, 3233-3241 (1997).
11. May, L. A., Smiley, B. & Schmidt, M. G. Comparative denaturing gradient gel electrophoresis analysis of fungal communities associated with whole plant corn silage. *Can. J Microbiol.* **47**, 829-841(2001).
12. Muyzer, G., De Waal, E. C. & Uitterlinden, A. G. Profiling of complex microbial populations by denaturing gradient gel electrophoresis analysis of polymerase chain reaction-amplified genes coding for 16S rRNA. *Appl. Environ. Microb.* **59**, 695-700 (1993).
13. Sanguinetti, M. C., Jiang, C., Curran, M. E. & Keating, M. T. A mechanistic link between an inherited and an acquird cardiac arrthytmia: HERG encodes the I Kr potassium channel. *Cell* **81**, 299-307 (1995).
14. Fierer, N. & Jackson, R. B. The diversity and biogeography of soil bacterial communities. *Proc. Natl. Acad. Sci. USA* **103**, 626–631 (2006).
15. Stemmer, M., Gerzabek, H. & Kandeler, E. Organic matter and enzyme activity in particle-size fractions of soils obtained after low-energy sonication. *Soil Biol.*

Biochem. **30**, 9–17 (1998).

16. Sessitsch, A., Weilharter, A., Gerzabek, M. H., Kirchmann, H. & Kandeler, E. Microbial Population Structures in Soil Particle Size Fractions of a Long-Term Fertilizer Field Experiment. *Appl. Environ. Microbiol*. **67**, 4215–4224 (2001).
